# Comment on “Berberine and Its Emerging Benefits in Psychiatric Conditions Especially Alzheimer's Disease”

**DOI:** 10.1155/2013/612684

**Published:** 2013-04-28

**Authors:** Shailendra Kapoor

**Affiliations:** Mechanicsville Hospital, Mechanicsville, VA 23223, USA

I read with great interest the recent paper by Ji and Shen [1]. Berberine may be of considerable benefit in a number of psychiatric disorders especially in Alzheimer's disease. 

Berberine inhibits the activation of nuclear factor kappaB by blocking the “mitogen-activated protein kinase” pathway [2]. Simultaneous inhibition of the phosphoinositide 3-kinase/protein kinase B pathway is also seen. Tau phosphorylation is also significantly attenuated. As a consequence, amyloid-beta peptide-stimulated production of IL-6 is markedly attenuated resulting in a significant reduction in neuroinflammation. Besides this, berberine markedly attenuates glycogen synthase kinase-3 activity [3]. Berberine also has an attenuating effect on the C-terminal fragments of amyloid precursor protein, thereby ultimately reversing learning defects in animal models. Berberine also activates the ERK1/2 pathway, thereby attenuating the expression of beta secretase which in turn further decreases the beta-amyloid production [4, 5]. Similarly, berberine exerts antidepressant effects in animal models. It mediates this function by altering intracranial dopamine, serotonin, and norepinephrine levels [6]. Its antidepressant effect is also mediated by and modulation of the nitric oxide pathway. 

The above examples clearly illustrate the emerging role of berberine in psychiatry and the need for further studies in this regard.
